# Supplementary figures and images for: Inhibition of Heparanase Expression Results in Suppression of Invasion, Migration and Adhesion Abilities of Bladder Cancer Cells
Source: Int J Mol Sci. 2020 May 27;21(11):3789. doi: 10.3390/ijms21113789 (PMC7313018; doi:10.3390/ijms21113789)

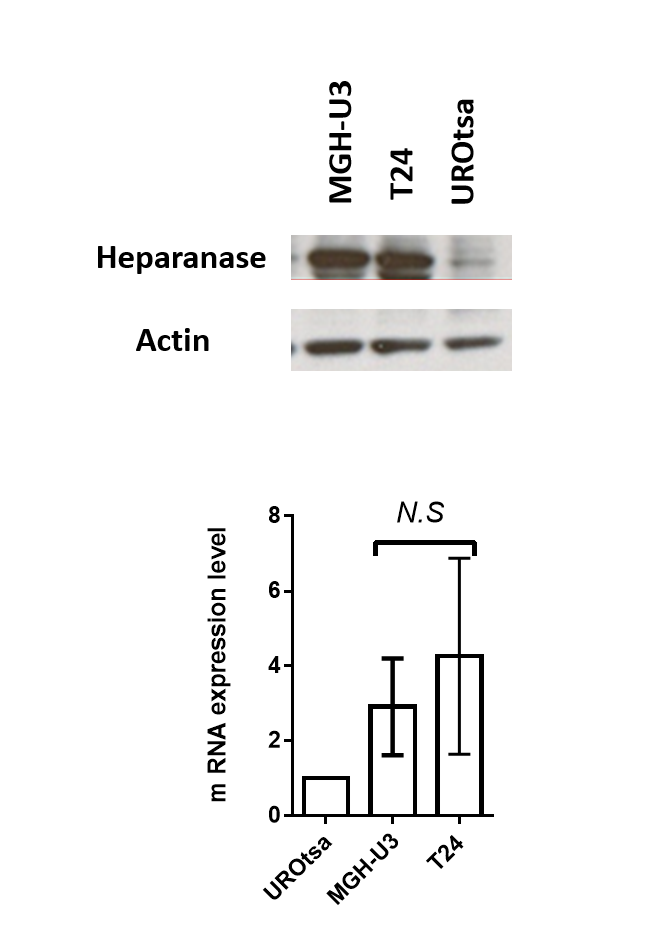

Supplement: Supplementary file 1 [file ijms-21-03789-s001.zip › Supplementary figure 1.tif]

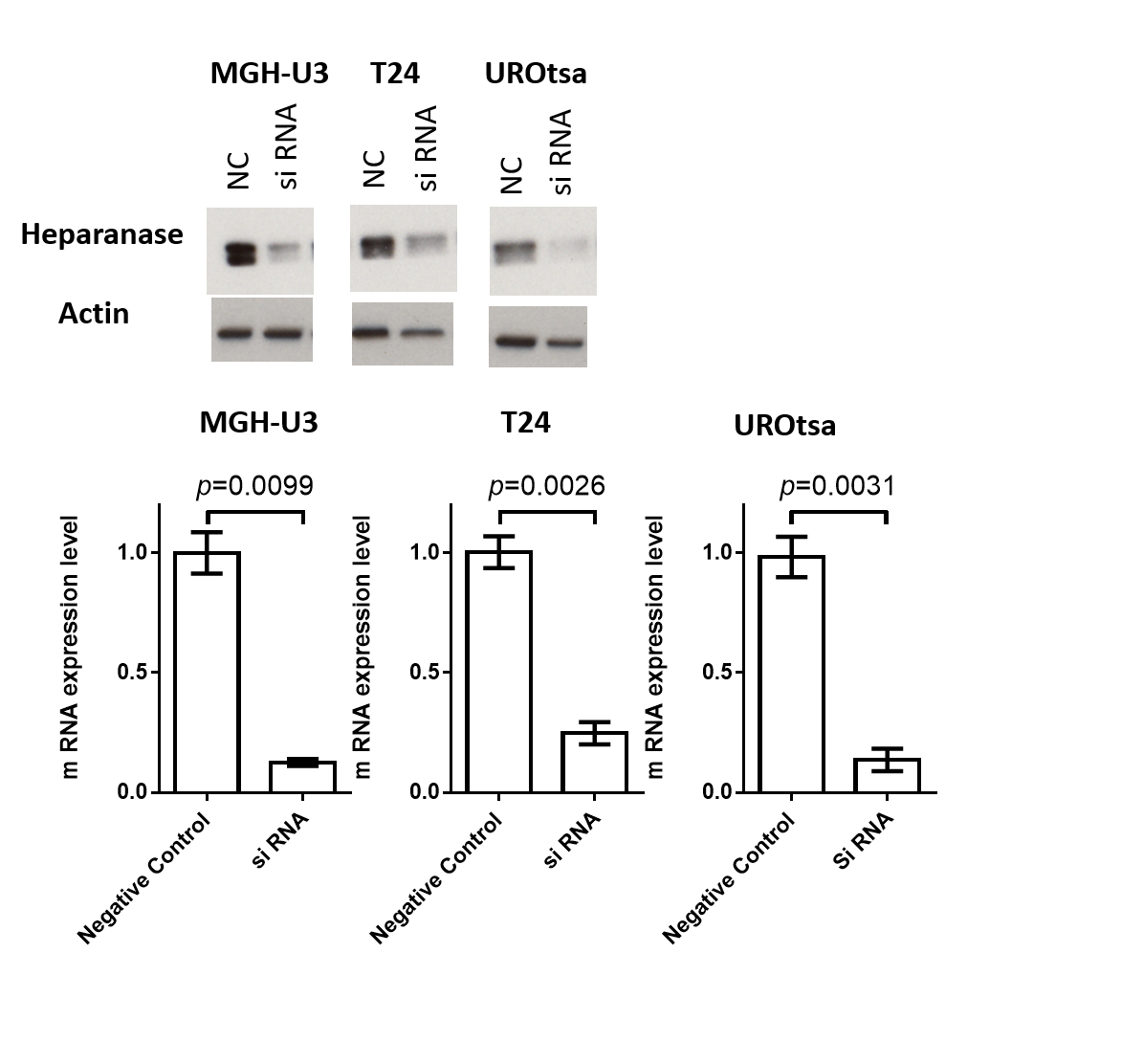

Supplement: Supplementary file 1 [file ijms-21-03789-s001.zip › Supplementary figure 2.tif]

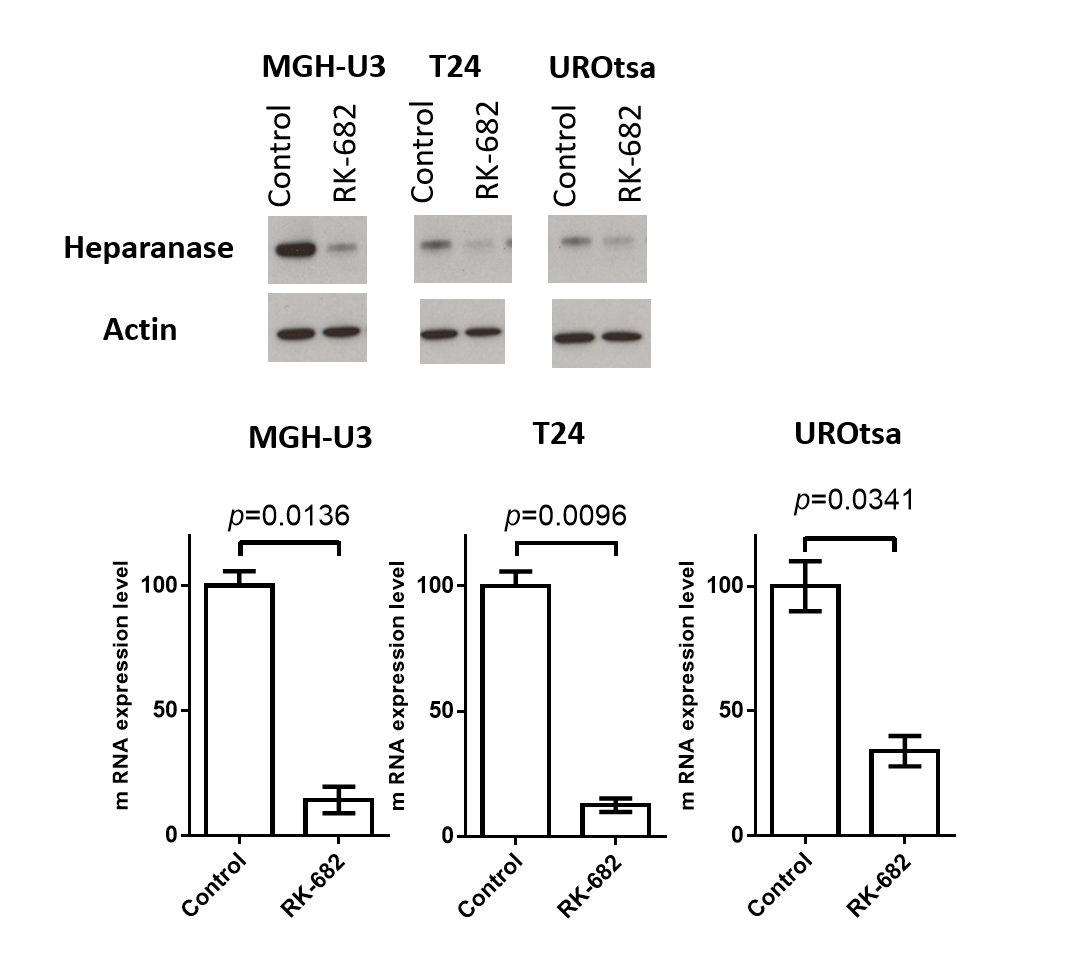

Supplement: Supplementary file 1 [file ijms-21-03789-s001.zip › Supplementary figure 3.tif]
